# Sex differences for clinical correlates of substantia nigra neuron loss in people with Lewy body pathology

**DOI:** 10.1186/s13293-024-00583-6

**Published:** 2024-01-19

**Authors:** Ece Bayram, David G. Coughlin, Ravi Rajmohan, Irene Litvan

**Affiliations:** 1 Department of Neurosciences, Parkinson and other Movement Disorders Center, University of California San Diego, 9500 Gilman Dr, La Jolla, CA 92093-0886 USA; 2 Department of Neurology, University of California Irvine, 1001 Health Sciences Road, Irvine, CA 92697-3950 USA

**Keywords:** Substantia nigra, Sex, Lewy body, Clinicopathological correlations

## Abstract

**Background:**

Lewy body dementia (LBD) phenotype is associated with the presence and degree of Lewy body, Alzheimer’s pathologies, and substantia nigra neuron loss. Nigral neuron loss is associated with parkinsonism in LBD, and females with LBD are less likely than males to have parkinsonism. As sex differences were reported for clinical correlates of Lewy body and Alzheimer’s pathologies, we aimed to investigate whether there are also sex differences for correlates of nigral neuron loss.

**Methods:**

Data were obtained from the National Alzheimer’s Coordinating Center for females (*n* = 159) and males (*n* = 263) with brainstem, limbic, and neocortical Lewy body pathology. Sex differences for the nigral neuron loss’ association with Lewy body pathology staging and core clinical LBD features (cognitive fluctuations, visual hallucinations, rapid eye movement sleep behavior disorder, parkinsonism) during follow-up were analyzed with generalized linear models adjusting for age and Alzheimer’s pathology staging. Whether any of the core clinical features at the time of dementia onset can predict underlying nigral neuron loss for females and males were also analyzed with generalized linear models.

**Results:**

Compared to males, females died older and had higher levels of Braak tau staging, but had similar levels of Lewy body pathology staging and nigral neuron loss. Females were less likely than males to have a clinical Lewy body disease diagnosis during follow-up. More advanced Lewy body pathology staging was associated with more nigral neuron loss, more so for males than females. More nigral neuron loss was associated with parkinsonism and clinical LBD diagnosis during follow-up, more so for males than females. Across the subgroup with dementia (40 females, 58 males), core LBD features at first visit with dementia were not associated with nigral neuron loss.

**Conclusions:**

Nigral neuron loss’ association with Lewy body pathology staging and core LBD features can differ by sex. Compared to males, females with Lewy body pathology have a higher risk of underdiagnosis. There is a need to elucidate the mechanisms underlying sex differences for pathology and clinicopathological correlations to advance diagnostic and therapeutic efforts in LBD.

## Highlights

- Clinical and pathological correlates of nigral loss differ by sex in people with brainstem, limbic and neocortical Lewy body pathology from the NACC.
- Compared to males with similar age and Alzheimer’s co-pathology, females have less nigral loss at brainstem and limbic Lewy body pathology stages.
- Compared to males with similar age, Lewy body and Alzheimer’s pathology staging, nigral loss association with parkinsonism and Lewy body dementia phenotype is weaker in females.
- Clinical features at first visit with dementia may not be associated with underlying nigral loss.
- Parkinsonism during follow-up may suggest underlying nigral loss for males, but perhaps not as reliably for females.

## Abstract (plain language summary)

Lewy body dementia (LBD) is the third most common dementia associated with Lewy body pathology, Alzheimer’s pathology, and substantia nigra loss. It is often less recognized in females compared to males, because the typical symptoms are less evident in females. In this study, we investigated whether substantia nigra neuron loss plays a role in the atypical presentation of LBD in females, contributing to the underdiagnosis compared to males. We analyzed data from 159 females and 263 males with pathological Lewy body disease obtained from the National Alzheimer's Coordinating Center. Females tended to be older at the time of death and had more tau buildup, but similar levels of Lewy body pathology and substantia nigra neuron loss compared to males. When we compared males and females of similar age with similar levels of Alzheimer's pathology, we observed that females had less substantia nigra neuron loss at less advanced Lewy body pathology stages. Greater nigral neuron loss was associated with parkinsonism and the typical LBD symptoms in males, but not as strongly in females. The extent of nigral loss could not be predicted based on the clinical features at the time of dementia diagnosis. Thus, the relationship between nigral neuron loss and the LBD symptoms seems to vary by sex. Females with underlying Lewy body disease are more likely to be underdiagnosed compared to males. We need further work to understand why these sex differences exist and how we can better identify and treat LBD.

## Background

Lewy body pathology is associated with prevalent and burdensome neurodegenerative Lewy body diseases such as Parkinson’s disease (PD) and dementia with Lewy bodies (DLB) [1]. Lewy body dementia (LBD) is an umbrella term including PD dementia (PDD) and DLB, which have substantial overlap for pathology and clinic [2]. Due to these overlaps, differentiation of PD, PDD, and DLB may not always be possible in pathologically defined cohorts with limited clinical information. Typical LBD phenotype consists of a combination of cognitive fluctuations, recurrent visual hallucinations, REM sleep behavior disorder, and parkinsonism [2–4]. Currently, the definite diagnosis of LBD can only be made by neuropathological examination [5]. Accurate in vivo diagnosis of LBD can be challenging due to clinical heterogeneity which in part is related to pathological heterogeneity, such as the degree of Lewy body pathology, nigral neuron loss, and the presence and degree of frequently co-occurring Alzheimer’s disease (AD) neuropathological changes [6]. Substantia nigra neuron loss is among the pathological hallmarks of PD and also occurs in DLB although possibly with less severity [7]. In DLB, substantia nigra neuron loss, higher levels of Lewy body pathology, and lower levels of AD co-pathology are associated with parkinsonism and an increased likelihood of a typical phenotype [5].

Sex is associated with different prevalence and clinical correlates for both Lewy body and AD pathologies [8–11]. Clinical diagnostic accuracy for LBD may be lower for females compared to males [12]. Neocortical Lewy body pathology is more common in males; AD co-pathology is more common in females [8, 9, 13]. AD co-pathology is associated with a lower likelihood of a typical LBD phenotype including lower likelihood for cognitive fluctuations, visual hallucinations, REM sleep behavior disorder, and parkinsonism, for both females and males [10]. In those with similar levels of Lewy body pathology staging with or without AD co-pathology, females are less likely to have dementia or have LBD phenotype including a lower likelihood for cognitive fluctuations, visual hallucinations, REM sleep behavior disorder, and parkinsonism [10, 11]. These findings highlight the sex differences in the clinicopathologic presentation of LBD. Investigating sex-specific differences in both the pathological and clinical presentations of LBD may not only improve clinical diagnostic accuracy but may lead to discoveries about the disease pathogenesis.

The lower prevalence of parkinsonism in females compared to males [12], may suggest an underlying difference for nigral neuron loss. However, sex differences for clinicopathological correlations of nigral loss have not been investigated in LBD. To address this gap, we investigated the correlation between core clinical LBD features and the degree of nigral loss in females and males with Lewy body pathology using the large pathologically validated dataset from the National Alzheimer’s Coordinating Center (NACC). We assessed potential sex differences for the association between Lewy body pathology and substantia nigra neuron loss levels and also analyzed whether any of the core clinical features at the time of dementia onset can help predict underlying nigral neuron loss.

## Methods

### Participants and measures

Data were obtained from the NACC Uniform Data Set (UDS) and Neuropathology Data Set for visits conducted between September 2005 and August 2019 at 39 past and present AD Research Centres [14–17]. Data collection was approved by local Institutional Review Boards of all contributing centres and informed consents were obtained from the participants prior to participation.

Data are collected by trained clinic personnel and clinicians using a standardized evaluation. We included participants with Lewy body pathology (brainstem, limbic, neocortical) [18], available substantia nigra neuron loss, and AD pathology staging data independent of the cognitive state or clinical diagnosis. Participants with other pathologic diagnoses associated with cognition were excluded (i.e., hippocampal sclerosis, multiple system atrophy, frontotemporal degeneration, traumatic brain injury, infections, other tauopathies, trinucleotide repeat diseases). These criteria provided a sample of 159 females and 263 males from 30 past and present AD Research Centres in the NACC for our analysis.

Clinician report of core LBD features (cognitive fluctuations, visual hallucinations, REM sleep behavior disorder, and parkinsonism), cognitive state, and clinical diagnosis at the last visit before death; the presence of core LBD features during follow-up were included. For people with a dementia diagnosis, core LBD features and clinical diagnosis at first visit with dementia were also included. Cognitive state ranged between (1) normal cognition, (2) impaired but not mild cognitive impairment (MCI), (3) MCI, and (4) dementia. CDR® Dementia Staging Instrument–Sum of Boxes (CDR®–SOB) for dementia severity and Neuropsychiatric Inventory-Questionnaire (NPI-Q) for behavioral symptom severity were included. Clinical diagnoses were made by the clinicians based on the available diagnostic criteria at the date of examination. Pathology variables included Lewy body pathology stage (brainstem-predominant, limbic, neocortical), NIA-AA AD neuropathologic change score [19], Thal phase, Braak tau stage, CERAD neuritic plaque score, substantia nigra neuron loss level (none, mild, moderate, severe). Lewy body pathology staging is consistent with Lewy pathology consensus criteria, which has high inter-rater reliability [18]. Classification is based on the presence/absence of Lewy bodies in specified regions. Diagnosis of the brainstem, limbic, or neocortical Lewy body indicates at least sparse Lewy bodies or Lewy neurites in any of the regions that were assessed.

### Statistical analysis

IBM SPSS Version 28.0 (Armonk, New York, USA) and R Version 4.2.3 [20] were used for statistical analysis. Demographics, clinical features, and scale scores were compared between females and males with χ^2^ and *t* tests, as appropriate. Cognitive state and neuropathological features were compared between females and males with Mann–Whitney *U* tests, and generalized linear models including age as a covariate. Sex differences for the association between Lewy body pathology staging and nigral neuron loss were performed by generalized linear mixed models adjusting for age at death, Thal phase, Braak tau stage and CERAD score. Sex differences for the association between substantia nigra neuron loss levels and clinical features were performed by generalized linear mixed models adjusting for age at the last visit, Lewy body, and AD pathology staging. In the subgroup of people without dementia at baseline who were diagnosed with dementia during follow-up, the association between substantia nigra neuron loss levels and clinical features at first visit dementia, changes in CDR^®^–SOB and NPI-Q scores after the first visit with dementia were assessed with generalized linear mixed models. Sex-stratified generalized linear models were performed to determine which core clinical features (cognitive fluctuations, visual hallucinations, REM sleep behavior disorder, parkinsonism) can predict underlying substantia nigra neuron loss. Alpha level < 0.05 was considered statistically significant. False discovery rate correction was used for multiple comparisons, and corrected *p* values are reported.

## Results

Demographics, clinical features, and neuropathological variables for females and males are shown in Tables 1 and 2. Females were, on average, older at baseline and last visit, and died older than males. The majority of the cohort identified as non-Hispanic and White. Years of education, follow-up duration, interval between the last visit and death were similar for females and males. At the last visit, CDR®–SOB scores and overall cognitive state were similar for females and males. Males had higher NPI-Q scores at the last visit and experienced cognitive decline at a younger age. Overall Lewy body and AD pathology staging and level of substantia nigra neuron loss were similar for females and males. Compared to males, females had higher levels of tau pathology by Braak tau staging.

**Table 1:** Demographics and clinical features of whole cohort

	Females (*n* = 159)	Males (*n* = 263)	*p* value
Age at baseline visit	75.0 (9.81)	72.7 (8.62)	0.040*
Years of education	16.9 (13.5)	16.5 (5.97)	0.73
Ethnicity, Hispanic (%)	3 (1.9%)	9 (3.4%)	0.73
Race (%)			0.1
-Asian	2 (1.3%)	0 (0%)	
-Black or African American	11 (6.9%)	7 (2.7%)	
-White	146 (91.8%)	254 (96.6%)	
Age at last visit	79.6 (10.7)	76.9 (9.34)	0.023*
Age at death	81.6 (10.2)	78.7 (9.30)	0.017*
Follow-up duration, years	4.63 (3.65)	4.16 (2.93)	0.22
Age at cognitive decline onset	70.8 (10.9)	68.3 (9.45)	0.040*
Cognitive state at baseline visit (%)			0.023*
-Normal cognition	35 (22.0%)	21 (8.0%)	
-Cognitively impaired but not MCI	3 (1.9%)	3 (1.1%)	
-MCI	32 (20.1%)	67 (25.5%)	
-Dementia	89 (56.0%)	172 (65.4%)	
Cognitive state at last visit (%)			0.058
-Normal cognition	18 (11.3%)	12 (4.6%)	
-Cognitively impaired but not MCI	5 (3.1%)	5 (1.9%)	
-MCI	13 (8.2%)	22 (8.4%)	
-Dementia	123 (77.4%)	224 (85.2%)	
CDR^®^–SOB at last visit	10.2 (6.90)	10.8 (6.09)	0.5
NPI-Q at last visit	5.38 (5.50)	7.70 (6.88)	0.005*
Clinical diagnosis of Lewy body disease (%)	41 (25.8%)	113 (43.0%)	0.005*
-Parkinson’s disease	16 (10.1%)	55 (20.9%)	0.017*
-Parkinson’s disease dementia	9 (5.7%)	41 (15.6%)	0.017*
-Dementia with Lewy bodies	25 (15.7%)	58 (22.1%)	0.18
Clinical diagnosis of AD (%)	109 (68.6%)	191 (72.6%)	0.5
Cognitive fluctuations during FU (%)	44 (32.6%)	109 (44.3%)	0.058
Visual hallucinations during FU (%)	61 (38.6%)	105 (40.1%)	0.82
REM sleep behavior disorder during FU (%)	29 (21.6%)	97 (39.9%)	0.005*
Parkinsonism during FU (%)	71 (47.3%)	156 (60.9%)	0.024*
Interval between last visit and death, months	24.5 (25.2)	22.6 (24.1)	0.55

All variables are reported as mean (standard deviation) or count (percentage). Group comparisons were performed using χ^2^, *t* and Mann–Whitney *U* (cognitive state) tests, as appropriate. Statistical significance is bolded and marked with * for FDR-corrected *p* < .05

*AD* Alzheimer’s disease, *CDR*^*®*^*–SOB* CDR^®^ Dementia Staging Instrument–Sum of Boxes, *FU* follow-up, *MCI* mild cognitive impairment, *NPI-Q* Neuropsychiatric Inventory-Questionnaire

**Table 2:** Neuropathological features of whole cohort

	Females (*n* = 159)	Males (*n* = 263)	*p* value
Postmortem interval, hours	20.3 (27.6)	19.9 (26.5)	0.91
Lewy body pathology stage (%)			0.92
-Brainstem-predominant	15 (9.4%)	30 (11.4%)	
-Limbic (transitional)	57 (35.8%)	85 (32.3%)	
-Neocortical (diffuse)	87 (54.7%)	148 (56.3%)	
Level of substantia nigra neuron loss (%)			0.068
-None	28 (17.6%)	38 (14.4%)	
-Mild	58 (36.5%)	82 (31.2%)	
-Moderate	49 (30.8%)	78 (29.7%)	
-Severe	24 (15.1%)	65 (24.7%)	
Alzheimer’s disease neuropathologic change (%)			0.084
-None	5 (3.1%)	20 (7.6%)	
-Low	20 (12.6%)	41 (15.6%)	
-Intermediate	39 (24.5%)	66 (25.1%)	
-High	94 (59.1%)	133 (50.6%)	
Thal amyloid phase	4.11 (1.31)	3.81 (1.55)	0.088
Braak tau stage	4.72 (1.58)	4.31 (1.65)	0.017*
CERAD neuritic plaque score	2.20 (1.06)	2.12 (1.08)	0.5
Presence of ischemic, hemorrhagic or vascular pathology (%)	158 (99.4%)	261 (99.6%)	0.8

All variables are reported as mean (standard deviation) or count (percentage). Group comparisons were performed using Mann–Whitney *U* and *t* tests (for interval), as appropriate. Statistical significance is bolded and marked with * for FDR-corrected *p* < 0.05

Adjusted for age at last visit, the overall cognitive state continued to be similar for females and males (*p* = 0.19). Adjusted for age at death, Lewy body pathology, nigral loss, and CERAD neuritic plaque level were similar for females and males (*p* = 0.79, *p* = 0.10, *p* = 0.11, respectively). Compared to males, females had higher levels of Thal amyloid and Braak tau stage (*p* = 0.018, *p* = 0.002, respectively).

Females were less likely than males to receive clinical PD and PDD diagnoses during follow-up. Similar percentages of females and males had clinical AD or DLB diagnosis at the last visit or during follow-up. Males were more likely than females to be reported to have REM sleep behavior disorder and parkinsonism. Similar ratios of females and males were reported to have cognitive fluctuations and visual hallucinations at the last visit.

Out of 73 females and 91 males without dementia at baseline, 40 females (54.8%) and 58 males (63.7%) had dementia diagnosis by the last visit. For this subgroup of females and males, their clinical features at first visit with dementia and neuropathological features were similar (Table 3).

**Table 3:** Features of people who did not have a dementia diagnosis at baseline and were diagnosed with dementia during follow-up

	Females (*n* = 40)	Males (*n* = 58)	*p* value
Clinical features at first visit with dementia			
Age	80.4 (8.96)	77.9 (10.6)	0.42
Years of education	16.9 (13.5)	16.6 (3.08)	0.9
CDR^®^–SOB	6.83 (5.69)	5.54 (3.76)	0.36
NPI-Q	4.82 (5.55)	5.35 (5.04)	0.81
Clinical diagnosis of Lewy body dementia (%)	7 (17.5%)	20 (34.5%)	0.27
-Parkinson’s disease dementia	3 (7.5%)	7 (12.1%)	0.69
-Dementia with Lewy bodies	4 (10.0%)	13 (22.4%)	0.27
Clinical diagnosis of AD (%)	31 (77.5%)	42 (72.4%)	0.79
Cognitive fluctuations (%)	7 (21.2%)	16 (30.2%)	0.59
Visual hallucinations (%)	7 (17.9%)	11 (19.0%)	0.9
REM sleep behavior disorder (%)	2 (5.9%)	13 (24.5%)	0.15
Parkinsonism (%)	7 (18.4%)	26 (46.4%)	0.072
Neuropathological features			
Lewy body pathology stage (%)			0.83
-Brainstem-predominant	2 (5.0%)	7 (12.1%)	
-Limbic (transitional)	14 (35.0%)	17 (29.3%)	
-Neocortical (diffuse)	24 (60.0%)	34 (58.6%)	
Level of substantia nigra neuron loss (%)			0.89
-None	8 (20%)	9 (15.5%)	
-Mild	10 (25.0%)	17 (29.3%)	
-Moderate	13 (32.5%)	18 (31.0%)	
-Severe	9 (22.5%)	14 (24.1%)	
AD neuropathologic change (%)			0.27
-None	0 (0%)	4 (6.9%)	
-Low	7 (17.5%)	4 (6.9%)	
-Intermediate	6 (15.0%)	23 (39.7%)	
-High	27 (67.5%)	27 (46.6%)	
Thal amyloid phase	4.43 (0.98)	3.79 (1.41)	0.072
Braak tau stage	4.75 (1.69)	4.45 (1.39)	0.27
CERAD neuritic plaque score	2.38 (1.01)	2.17 (0.94)	0.27
Presence of ischemic, hemorrhagic or vascular pathology (%)	40 (100%)	58 (100%)	N/A

All variables are reported as mean (standard deviation) or count (percentage). Group comparisons were performed using χ^2^, Mann–Whitney *U* and *t* tests, as appropriate. Statistical significance is bolded and marked with * for FDR-corrected *p* < 0.05

*AD* Alzheimer’s disease, *CDR*^*®*^*–SOB* CDR^®^ Dementia Staging Instrument–Sum of Boxes, *NPI-Q* Neuropsychiatric Inventory-Questionnaire, *N/A* not applicable

### Sex differences for substantia nigra neuron loss associations

**Fig. 1:**
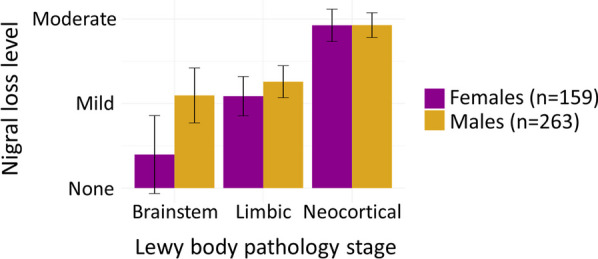
Association between Lewy body pathology stage and nigral neuron loss. Figure depicts the model adjusted for age at death, Thal amyloid phase, Braak tau stage and CERAD neuritic plaque score

There were significant sex differences for Lewy body pathology associations with nigral neuron loss (*p* < 0.001) (Fig. 1). Higher level of Lewy body pathology staging was associated with more substantia nigra neuron loss for males (*B* = 1.29, *p* < 0.001) compared to females (*B* = 1.24, *p* < 0.001). This was largely driven by differences in nigral neuron loss observed in brainstem and limbic stage Lewy pathology.

**Fig. 2:**
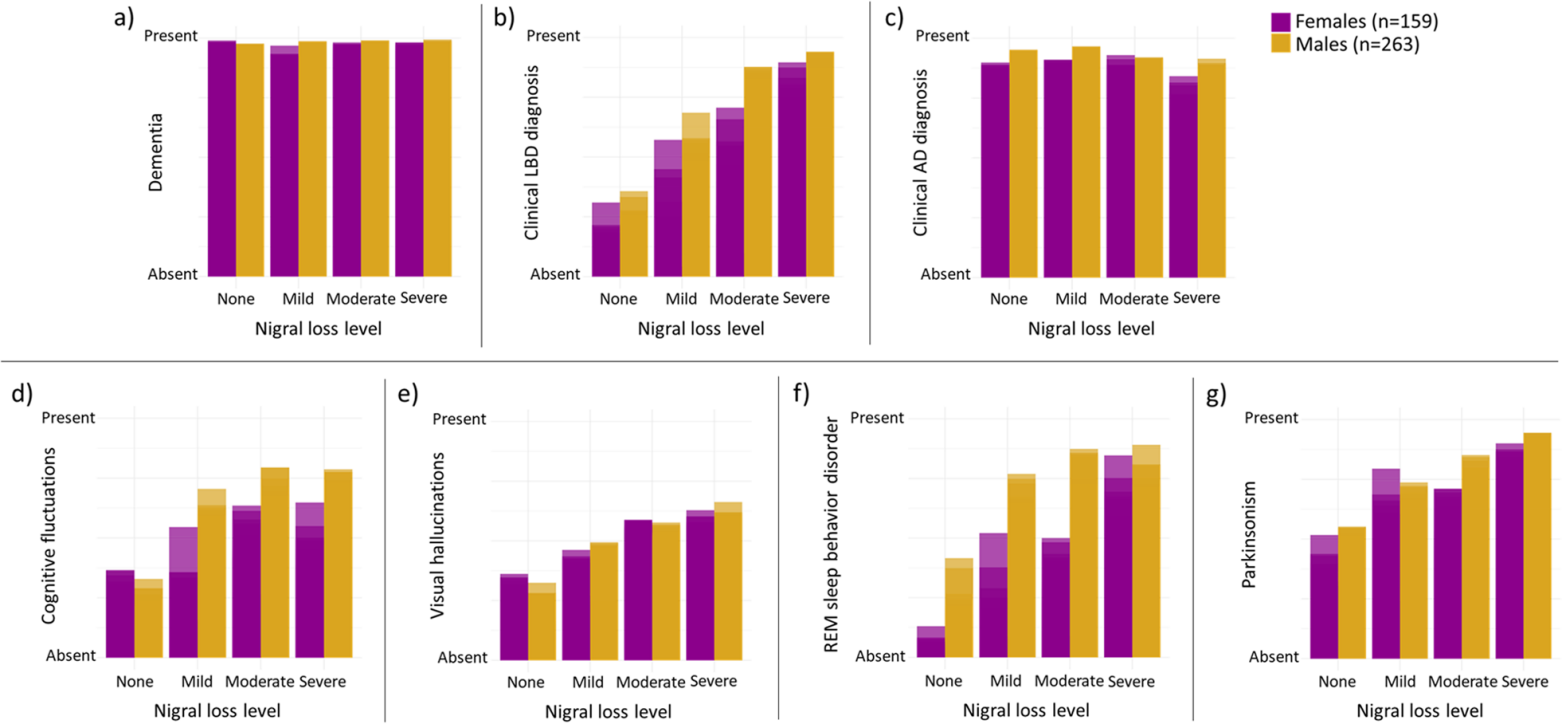
Nigral loss level associations with likelihood of clinical features during follow-up. Figure depicts the models adjusted for age at last visit, Lewy body pathology staging, Thal amyloid phase, Braak tau stage, and CERAD neuritic plaque score. *AD* Alzheimer’s disease, *LBD* Lewy body dementia

In longitudinal outcomes adjusting for Lewy body and AD pathology staging, nigral neuron loss was not associated with dementia likelihood (*β* = 0.13, *p* = 0.46), but was associated with a higher likelihood for a Lewy body disease clinical diagnosis (*β* = 0.75, *p* < 0.001) and lower likelihood for AD diagnosis (*β* = − 0.30, *p* = 0.038). More nigral loss was associated with higher Lewy body disease diagnosis likelihood more so in males (*β* = 0.56 for females; *β* = 0.85 for males; sex interaction *p* < 0.001) and lower AD diagnosis likelihood more so for females (β = − 0.47 for females; β = − 0.17 for males; sex interaction *p* = 0.022) (Fig. 2).

In the subgroup with a dementia diagnosis during follow-up adjusting for Lewy body and AD pathology staging, nigral neuron loss was not associated with Lewy body disease or AD clinical diagnosis, core clinical features, CDR^®^–SOB or NPI-Q scores at first visit with dementia; or change of CDR^®^–SOB and NPI-Q scores over time after the first visit with dementia (*p* > 0.064 for all).

### Clinical predictors for underlying substantia nigra neuron loss for females and males

In unadjusted models, parkinsonism during follow-up for males (*β* = 1.26, *p* < 0.001), and parkinsonism and RBD during follow-up for females were associated with more nigral loss (*β* = 0.78, *p* = 0.027; *β* = 1.18, *p* = 0.007). Once models were adjusted for Lewy body pathology and AD pathology staging, parkinsonism remained associated for males (*β* = 0.98, *p* < 0.001), but neither parkinsonism nor RBD was associated for females (*β* = 0.69, *p* = 0.055; *β* = 0.80, *p* = 0.079).

In the subgroup with a dementia diagnosis during follow-up, none of the core clinical features at the first visit with dementia significantly predicted underlying nigral loss in unadjusted (*p* > 0.059 for all) or adjusted models including Lewy body pathology and AD pathology staging (*p* > 0.12 for all).

## Discussion

In this study, we investigated clinical differences between females and males associated with substantia nigra neuron loss in people with brainstem, limbic, or neocortical Lewy body pathology in the NACC dataset. Following up on our prior reports on the sex differences for clinical correlates of Lewy body and AD pathologies [10, 11], we hypothesized that more nigral loss would have a stronger association with LBD phenotype in males compared to females. Our analyses showed that despite similar levels of nigral loss for females and males in our cohort, clinical correlates and pathological associations were significantly different by sex. Nigral loss’ association with Lewy body pathology staging, parkinsonism, and LBD phenotype were significantly stronger in males. During the follow-up period for the whole cohort, parkinsonism was a significant predictor for underlying nigral loss only for males after accounting for accompanying Lewy body and AD pathologies. These findings support previous reports on nigral loss association with parkinsonism, PD, and LBD phenotype [4, 21], and also indicate that the association differs by sex.

Substantia nigra neuron loss is one of the pathological hallmarks of PD pathology, although it may not always occur in DLB [22]. As nigral loss is associated with parkinsonism, it plays a central role in PD; not all people with DLB experience parkinsonism and may not have nigral loss [4, 23]. Despite some reports on higher levels of Lewy body pathology burden correlating with more nigral loss, the association between nigral neuron loss and Lewy body pathology is not straightforward as Lewy bodies may be cleared from dead nigral cells after some time, leading to conflicting findings [24, 25]. Nigral neuronal loss can also occur and correlate with parkinsonism in the absence of Lewy body pathology [22, 26–28]. Previous studies have shown that nigral neuron response to Lewy body disease can differ by sex with different pathways being activated in females and males. Compared to females, males can have selective nigral neuron loss without robust astrocytosis [29] and upregulation of genes related to PD (*SNCA, PINK1*) in dopaminergic neurons [30]. Our findings suggest males may be more vulnerable than females to nigral neuronal loss in association with lower stages of Lewy body pathology (brainstem, limbic). Disease mechanisms contributing to nigral neuron loss may be triggering different responses in females and males with Lewy body pathology. Our findings revealed sex-based differences in the associations of core clinical features to nigral loss in the setting of Lewy body pathology. This may contribute to the lower likelihood of parkinsonism in females with Lewy body pathology. Males with Lewy body pathology are more likely to have more nigral loss and consequently higher likelihood for parkinsonism and LBD phenotype [10, 11]. Both sex-specific clinical and pathologic correlates of Lewy body pathology may impact the clinical phenotype.

Females were less likely to have REM sleep behavior disorder, parkinsonism, and subsequently a clinical PD diagnosis during follow-up despite similar levels of nigral loss and Lewy body pathology staging compared to males. This finding is in line with previous reports on sex differences in PD prevalence and Lewy body disease features [12, 31]. Males reported cognitive decline at a younger age than females, which may be associated with the higher dementia risk for males with Lewy body disease, and also females with Lewy body disease under-reporting their symptoms and other social factors [31, 32]. Distinct brain regions may be impacted differently by these pathologies for females and males, and future work on the level of pathology for different brain areas is needed.

Compared to the male sex, the female sex may be associated with an increased risk for AD pathological changes [33]. Autopsy studies have shown higher tau staging in females compared to males [13, 34], which was also shown in this cohort. Older age has been associated with more tau burden in people with DLB and positive tau biomarkers (cerebrospinal fluid phosphorylated tau, AV-1451 PET) were associated with a lower likelihood for REM sleep behavior disorder and parkinsonism [35]. In our cohort, females had similarly higher levels of mean tau burden, were older, and had a lower likelihood for REM sleep behavior disorder and parkinsonism. Tau pathology is associated with cognitive decline in people with Lewy body pathology, with a stronger association for females than males [10]. However, findings in AD suggest a non-linear association between tau pathology and cognitive decline [36]. Females may bear higher levels of tau burden before developing cognitive impairment, with similar levels of impairment observed in females and males with Braak tau staging 5/6 [36]. In our cohort, females had a higher level of tau staging than males, yet a mean below 5, and were less likely to have dementia. On the other hand, the distribution of regional tau pathology in AD and LBD may differ, with some reports showing a relative sparing of medial temporal lobe structures [37, 38]. In AD, regional tau burden detected by ^18^ F-Flortaucipir PET differed by sex and sex was a mediating factor for the association between regional tau and cognitive decline [39]. Although models for nigral loss clinical correlates were adjusted for AD neuropathologic change, only traditional staging data were available for AD neuropathology and regional pathology impact could not be considered.

Cerebrovascular pathology may be common in people with Lewy body pathology and impact clinical outcomes [40]. Although over 99% of the individuals in our cohort had ischemic, hemorrhagic, or vascular pathologies, we were unable to evaluate the degree of these pathologies and their clinical correlates. Studies have suggested sex differences for the prevalence and clinical correlates of vascular risk factors and cerebrovascular pathologies in vascular dementia and AD [41, 42]. Sex hormones may contribute to these differences in prevalence and impact. Nevertheless, further research is still needed to determine the sex-specific impact of cerebrovascular pathologies in LBD.

In PD, nigral loss has been associated with disease progression and severity without sex differences in prior reports [21, 26]. This association in PD was also reported to be particularly strong in the first 5 years of symptomatic disease, slowly weakening afterward [21]. In the subgroup with dementia, we did not find any associations between nigral loss and severity of dementia or behavioral symptoms at first visit with dementia or over time. This negative finding may be due to only 10% of the subgroup with dementia having a diagnosis of PD, and dementia typically occurring later on in the disease progression for people with PD [43]. The severity of parkinsonism or age of onset for parkinsonism was not evaluated due to more than 50% missing data on these measures.

Across the subgroup of people who were diagnosed with dementia during the follow-up, none of the core clinical features at the first visit with dementia were associated with nigral loss. The sample size is small and the available information on the staging of pathologies at death may not correspond to the underlying pathology presence and staging at the time of dementia. These findings also indicate the importance of follow-up and the need to re-assess the clinical diagnosis with disease progression. With the advances in biomarkers that can help detect underlying pathologies during life and through the combined use of different biomarkers [44], a more accurate assessment can be performed. On the other hand, the sex-specific accuracy of biomarkers should be determined as our findings underscore sex differences for clinicopathological correlations.

There are several strengths and limitations in this study. Our findings revealed sex differences for clinical associations of nigral loss, which provides further insight into why females are less likely than males to clinically present with the typical LBD phenotype. For the analyses, we leveraged the detailed neuropathological and clinical data from the NACC dataset, which includes a fairly large number of pathologically confirmed individuals with clinical characterizations performed by experts. Although the NACC dataset provides a large cohort of people from different states in the US, the majority of the cohort identified as non-Hispanic, White, and had high levels of education. This limits the generalizability of these findings. Dementia likelihood differs based on ethnicity, race, culture, medical comorbidities, socioeconomic status, and other social determinants of health [45]. Furthermore, dementia is a clinical diagnosis relying on the history provided by the individual, a close relative, friend, or caregiver, neurologic exam, and neuropsychological testing [46]. The presence of core features including REM sleep behavior disorder, cognitive fluctuations, and visual hallucinations was based on self or care-partner report. REM sleep behavior disorder is likely underreported for females due to females having less violent dreams or less dream enacting behavior [47] despite similar levels of activity on electromyographic activity [48]. In addition, compared to males with PD, females with PD perceive more discrimination and feel they are not being heard by their healthcare professionals [32]. This may lead to women with PD being less likely to report their symptoms and can contribute to a lower likelihood of diagnosis. Sampling bias can also limit our findings as females are underrepresented in Lewy body disease research and may be less aware of the symptoms associated with Lewy body disease [31]. However, both community and clinical cohorts have reported that biological factors for sex differences including genetics and hormonal profiles, impact the prevalence, progression, and treatment response in Lewy body diseases [12, 31]. Sex is an important biological variable in Lewy body disease and more research to better understand the factors behind these differences is needed. Environmental factors, psychosocioeconomic factors, and the impact of health disparities can play a role in the differences between women and men, and also interact with biological factors to change the level or even the direction of the impact.

### Perspectives and significance

Our findings suggest that while parkinsonism may be a reasonable clinical surrogate for nigral neuron loss, it may not be as sensitive for females. The diagnostic utility of imaging markers, such as dopamine transporter single-photon emission computed tomography associated with nigral loss [49], may differ for females and males. Existing clinical diagnostic criteria may be skewed towards the detection of LBD in males, which may in part result from the lack of representation of females in Lewy body disease research. These biases in clinical criteria likely contribute to the underdiagnosis of LBD in females with Lewy body pathology. This leads us to emphasize the importance of identifying new core clinical criteria that adequately detect the presence of Lewy body pathology in females, rather than focusing on nigral neuron loss, as a strategy to improve diagnostic accuracy in females. To this end, there is a need to further investigate the mechanisms underlying different clinicopathological correlations in females and males.

## Conclusions

Compared to men with similar levels of Lewy body and AD pathology, females had less severe nigral neuron loss, lower likelihood of an LBD phenotype, and lower likelihood of parkinsonism associated with nigral loss. None of the existing core clinical criteria were associated with nigral neuron loss in females with Lewy body pathology and only parkinsonism was associated with nigral neuron loss in males. As clinical diagnostic criteria are updated and new biomarkers are established for clinical diagnosis, it is imperative to take sex differences and the representation of diverse populations into consideration.

## Data Availability

Data are available upon request to the NACC (https://naccdata.org/requesting-data/data-request-process).
